# Identifying and breaking barriers: Addressing disparities in the care of patients with gynecologic cancers

**DOI:** 10.1016/j.gore.2025.101799

**Published:** 2025-06-30

**Authors:** Bhavana Pothuri, Michele Muir, Jean Hurteau, John Farley, Michelle D.S. Lightfoot, Summer Dewdney, Tara Castellano, John K. Chan, Sharad Ghamande, Al Asante-Facey, Marina Stasenko, B.J. Rimel, Electra D. Paskett

**Affiliations:** a Perlmutter Cancer Center, NYU Langone, New York, NY, USA; b GSK, Collegeville, PA, USA; c Dignity Health Cancer Institute, St. Joseph Hospital and Medical Center, Phoenix, AZ, USA; d Rush University Medical Center, Chicago, IL, USA; e LSU Health Sciences Center, New Orleans, LA, USA; f Sutter Health, San Francisco, CA, USA; g Georgia Cancer Center, Augusta University, Augusta, GA, USA; h Memorial Sloan Kettering Cancer Center, New York, NY, USA; i Cedars-Sinai Medical Center, Samuel Oschin Comprehensive Cancer Institute, Los Angeles, CA, USA; j Division of Cancer Prevention and Control, Department of Medicine, College of Medicine and Comprehensive Cancer Center, The Ohio State University, Columbus, OH, USA

**Keywords:** Gynecologic malignancy, Healthcare disparity, Patient navigator, LGBTQAI+

## Abstract

**Background:**

Significant disparities exist in the care of patients with gynecologic malignancies. Higher incidences of gynecologic malignancies among underrepresented subpopulations (eg, racial, ethnic, and/or LGBTQAI+) and lack of representative enrollment within clinical trials have highlighted the need to improve healthcare equity. We aimed to identify barriers to equitable health care and clinical trial participation for specific diverse populations of patients with gynecologic malignancies and to identify potential solutions for overcoming these barriers.

**Methods:**

A series of 4 live and 3 asynchronous advisory boards facilitated by GSK was conducted between January 2023 and July 2024; live advisory boards were population specific. Gynecologic oncologists, health researchers, advanced practice providers, patients, and patient advocacy group representatives who worked with and/or were themselves members of the focus population participated. Insights were compiled and analyzed to identify barriers and potential solutions across and within populations.

**Results:**

Common barriers to equitable health care across all populations included cost, transportation, level of health literacy, and provider biases; 11 population-specific barriers were noted, with LGBTQAI+ patients described as facing the most barriers. Patient navigator involvement was identified as a feasible and highly impactful solution for breaking multiple barriers across various diverse populations.

**Conclusions:**

Most barriers to equitable health care were population specific, affirming the need for continued consultation and discussions with members of communities and with individuals to address specific barriers and enact effective solutions. Engagement of patient navigators was identified as an important way to improve disparities within the care of gynecologic malignancies across all underrepresented patients.

## Highlights

• An advisory board series was conducted to identify barriers and solutions to equitable health care in diverse populations.
• Barriers common to all populations included cost, transportation, lack of health literacy, and provider bias.
• Population-specific barriers were noted for each population, with the most barriers identified among LGBTQAI+ patients.
• Increased use of patient navigators was noted to be a solution that could reduce multiple barriers across all populations.

## Graphical abstract

**Figure:**
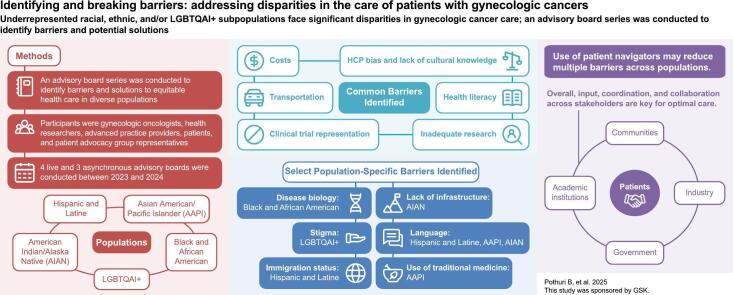

## 1 Introduction

Gynecologic malignancies are a significant burden in the United States (Siegel et al., 2024), with an estimated 116,930 new cases and 33,850 deaths in 2024 (Siegel et al., 2024). Despite this, gynecologic cancer research is significantly underfunded compared with other cancers, contributing to persistent unmet needs among patients (Zambrano et al., 2023). Even within gynecologic cancers, research funding is unevenly distributed, with funding for ovarian cancer research estimated to be 4 times higher than that for uterine cancer in 2025, despite similar and rising mortality rates for uterine cancer (Siegel et al., 2024, National Institutes of Health, 2025).

Incidence rates of cervical and uterine cancers are higher among Black and African American, American Indian/Alaska Native (AIAN), and Hispanic and Latine individuals compared with White individuals (Siegel et al., 2024, Melkonian et al., 2021). AIAN patients also have a higher incidence of ovarian cancer compared with White patients (Melkonian et al., 2021). Lesbian and bisexual individuals may have a higher risk of cervical cancer and a higher prevalence of certain risk factors for gynecologic cancers compared with heterosexual individuals (Quinn et al., 2015, Zaritsky and Dibble, 2010). Transgender individuals may also be at higher risk for certain cancers, including cervical cancer, compared with cisgender individuals (Leone et al., 2023).

The patient journey refers to the sequence of experiences a patient has with healthcare professionals (HCPs), spanning from the initial recognition of symptoms and risk factors through diagnosis, treatment, and aftercare, including survivorship. Health disparities are evident throughout the patient journey within gynecologic cancer care (Towner et al., 2022, Anderson et al., 2023, Rodriguez et al., 2021, Bristow et al., 2015). For example, Black and African American patients are more likely to be diagnosed with advanced stage endometrial cancer compared with White patients, contributing to lower 5-year survival rates (Black and African American, 63 % vs White, 84 %) and nearly double the mortality rate (9.1 per 100,000 population vs 4.6 per 100,000) (Siegel et al., 2024, Towner et al., 2022). Black and African American patients are also more likely to face delays in referrals to a gynecologic oncologist (Anderson et al., 2023). Black and African American, Hispanic and Latine, and AIAN patients are less likely to receive guideline-concordant care for endometrial cancer compared with Asian and White patients; the same is true for Black and African American patients with ovarian cancer (Rodriguez et al., 2021, Bristow et al., 2015).

Sexual and gender minorities, including lesbian, gay, bisexual, transgender, queer (or questioning), asexual (or allied), intersex (LGBTQAI+) individuals, also experience distinct challenges across all phases of care (Quinn et al., 2015, Leone et al., 2023, Ferreira-Filho et al., 2024, Wakefield, 2021, Boehmer, 2018, Alpert et al., 2022). Notably, some patients with gynecologic malignancies do not identify as women and may not feel welcome in spaces dedicated to “women’s cancers” (Alpert et al., 2022). Due to limited research, many of the driving factors of disparities in the LGBTQAI+ community are unknown (Quinn et al., 2015, Alpert et al., 2022).

There are also significant gaps in the representation of diverse populations within clinical trials, resulting in a lack of knowledge about the efficacy and safety of treatments within specific populations. Adjusted for population size, ovarian cancer trial enrollment was between 6- and 10-fold lower than expected among Black, Latine, and AIAN patients based on incidence rates (Richardson et al., 2023). Enrollment in uterine cancer trials was between 2.5- and 6-fold lower than expected among Latine and AIAN patients (Richardson et al., 2023).

Biologic and genetic differences among patients with different racial or ethnic backgrounds can result in differences in treatment options and efficacy (Towner et al., 2022, Webster et al., 2023, Eakin et al., 2023, Lee et al., 2023, Lara et al., 2024, Xu et al., 2023). Black and African American patients with uterine cancer were found to have fewer targetable mutations for treatment with immunotherapies, as well as mTOR and BRAF inhibitors, compared with White patients (Eakin et al., 2023), and may be more likely to have high-risk histologic subtypes (Lee et al., 2023, Lara et al., 2024). Black and African American patients with uterine cancer are also less likely than White patients to present with abnormal uterine bleeding and more likely to present with other symptoms, which may lead to delayed diagnosis (Xu et al., 2023). However, even after accounting for symptom presentation, a disparity in early diagnosis remains (Xu et al., 2023).

Structural factors such as racism, sexism, poverty, and other social determinants of health also contribute to disparities during the patient journey and in clinical trial enrollment. Barriers including socioeconomic status, language and communication issues, mistrust of HCPs, limited health literacy, and costs of care all contribute to unequal access to care. For example, a patient’s ability or knowledge to self-advocate may be impacted by health literacy and cultural beliefs (Chapman-Davis et al., 2022). Past experiences with discrimination and mistrust of the healthcare system lead many to avoid seeking care (Ferreira-Filho et al., 2024, Wakefield, 2021). Geospatial issues also exist, including neighborhood vulnerability and socioeconomic deprivation, in relation to healthcare access and the intersectionality of socioeconomic status with race (Gamble et al., 2022). Furthermore, investigator bias, lack of racial self-identification, stringent eligibility criteria, and language barriers have been factors for low trial enrollment of underrepresented racial, ethnic, and other groups (Alpert et al., 2022, Karpel et al., 2022, Karpel et al., 2022, Lara et al., 2023, Lee et al., 2022).

While every patient’s experience is unique, identifying barriers experienced by specific populations may be an important step to implement solutions with the potential to improve care for all patients. The objectives of this paper were to identify barriers to equitable health care for specific populations of patients with gynecologic malignancies throughout their patient journey, to explore ways to ensure and support diverse patients in clinical trial enrollment, and to develop best practices that emphasize the responsibility of HCPs, researchers, pharmaceutical companies, and patient advocacy groups in providing equitable care for those with gynecologic malignancies.

## 2 Materials and methods

A longitudinal engagement plan with 4 live and 3 asynchronous advisory board touchpoints was used (**Fig. S1**). This series was developed and supported by GSK and moderated by a gynecologic oncologist with leadership and expertise in health disparities and conducted between January 2023 and July 2024. Survey content for the asynchronous touchpoints was developed by a GSK team with diverse qualifications including but not limited to gynecologic oncology, pharmacy, and patient advocacy, with a focus on relevant congress abstracts and publications to facilitate data-driven dialogue (see Supplement for survey questions). The live advisory board content was developed by GSK in collaboration with the gynecologic oncologist expert who served as lead moderator for the live events. Participants in each touchpoint were compensated based on the number of hours spent on research/preparation, survey completion, and/or advisory board participation; all transfers of value were recorded in accordance with the Sunshine Act.

The live advisory board meetings were divided into sessions of moderated discussions and group workshops (**Fig. S2**). Advisors discussed barriers to equitable health care and clinical trial participation and completed interactive group activities to consolidate and rank proposed solutions, along with their impact and ease of implementation. The first live advisory board focused on general barriers and healthcare disparities among patients with gynecologic malignancies. Based on the outcomes from the first advisory board, 3 additional advisory boards were held that focused on specific diverse patient populations (eg, Black and African American, Hispanic and Latine, Asian American/Pacific Islander [AAPI], AIAN, LGBTQAI+).

Gynecologic oncologists, health researchers, advanced practice providers, patients, and patient advocacy group representatives who worked with and/or were themselves members of the focus population participated (**Fig. S3**). Between 7 and 12 participants were included in each live touchpoint.

Asynchronous advisory board touchpoints included gynecologic oncologists in the US. Each online touchpoint included a 13- or 15-question survey (see Supplement for survey questions). Each respondent had 2 h to complete the activity at their convenience over approximately 2 weeks, while still having the ability to interact with other advisors. An additional 6 or 7 gynecologic oncologists completed each online touchpoint.

Insights from all live and asynchronous touchpoints were compiled after the completion of the advisory board series and analyzed to identify barriers and potential solutions. Barriers and solutions were categorized based on theme into broad categories for quantification (eg, barriers related to transportation were all grouped together) and to identify similarities and differences across the populations; population-specific barriers were those identified, in the collective opinion of participants, to preferentially impact 1 or more of the specific patient populations discussed.

## 3 Results

### 3.1 Barriers to equitable health care

**Fig. 1:**
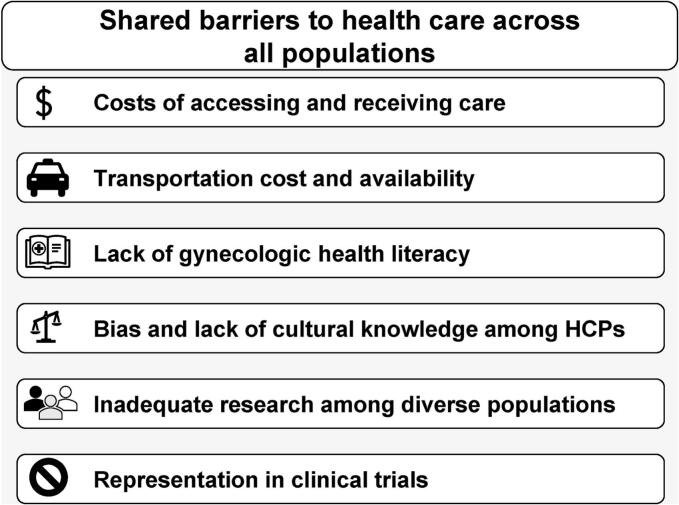
**Shared barriers to equitable health care across populations.** HCP, healthcare professional.

Overall, 22 distinct barriers to equitable health care were identified during the advisory board series, 6 of which were common across all populations (Fig. 1). Cost of accessing and/or receiving care and issues with transportation were identified as 2 of the most important barriers for all populations. Costs of care included costs related to treatment, insurance, missing work for appointments and treatments, and childcare. Transportation issues included availability of transportation, distance to appointments, and time needed for travel. Other common barriers included the patient’s level of health literacy, bias among HCPs, inadequate research among diverse populations, and lack of representation in clinical trials.

It should be noted that these common barriers may be experienced differently by individuals within the different populations. For example, advisors noted that bias among HCPs may result in delays in referrals or a lack of biomarker testing for Black and African American patients, while for LGBTQAI+ patients, provider biases and assumptions may cause patients to need to explain their pronouns, gender identity, or supportive partner multiple times during their care journey.

Five of the barriers identified were shared by multiple but not all populations (Table 1). Among Black and African American, Hispanic and Latine, AAPI, and AIAN populations, language was identified as an important barrier. Discrimination was noted among Black and African American, Hispanic and Latine, and LGBTQAI+ populations. Among Black and African American, AAPI, AIAN, and LGBTQAI+ populations, mistrust of healthcare systems and providers was identified as a barrier. A higher prevalence of chronic diseases among Black and African American and AIAN populations was also cited. Lack of representation among HCPs was noted in Black and African American and AAPI populations.

**Table 1:** Barriers to equitable health care in patients with gynecologic malignancies.

**Most common barriers**	**Populations impacted**
Language	• Hispanic and Latine • AAPI • AIAN

Discrimination and implicit bias	• Black and African American • Hispanic and Latine • LGBTQAI+

Mistrust of healthcare systems	• Black and African American • AAPI • AIAN • LGBTQAI+

Chronic diseases	• Black and African American • AIAN

Disease biology differences	• Black and African American

Immigration status	• Hispanic and Latine

Tendency to rely on family to obtain information and make treatment decisions	• Hispanic and Latine

Use of traditional medicines	• AAPI

Tendency to seek care only when health issues arise, limiting screening and cancer prevention behaviors	• AAPI

Belief system juxtaposing mainstream health care	• AIAN

Lack of infrastructure within the community	• AIAN

Lack of respect and safety	• LGBTQAI+

Stigma and lack of support	• LGBTQAI+

Denial of culturally appropriate care by the healthcare system	• LGBTQAI+

Lack of research on the impact of hormone therapy on cancer treatment	• LGBTQAI+

AAPI, Asian American and Pacific Islander; AIAN, American Indian and Alaska Native; HCP, healthcare professional; LGBTQAI+, lesbian, gay, bisexual, transgender, queer (or questioning), asexual (or allied), intersex.

Advisors also identified 11 population-specific barriers (Table 1). Population-specific barriers among the racial and ethnic populations included potential disease biology differences (Black and African American), immigration status (Hispanic and Latine), use of traditional medicines (AAPI), and juxtaposition of belief systems with mainstream healthcare (AIAN). The LGBTQAI+ population had the greatest number of population-specific barriers to equitable health care, with transgender and gender nonconforming individuals highlighted as facing the most barriers. Population-specific barriers among the LGBTQAI+ population included a lack of respect and safety for the patient and their partner, stigma, and lack of support during and after diagnosis.

**Fig. 2:**
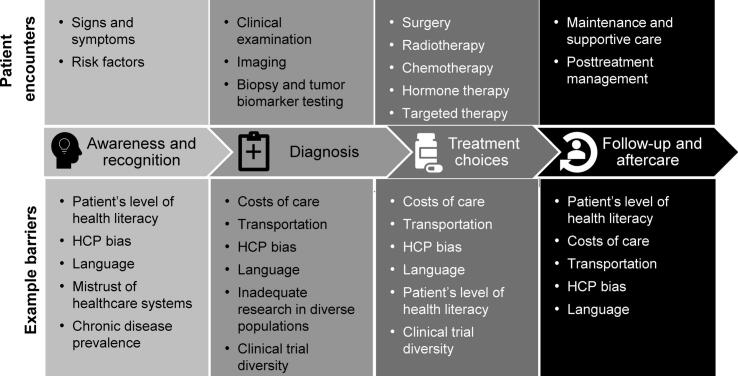
**Barriers encountered during the patient journey.** HCP, healthcare professional.

The barriers identified by the advisors impacted all phases of the patient journey (Fig. 2), with each barrier affecting multiple phases. For example, HCP biases affected awareness and recognition of symptoms and risk factors as well as diagnosis and treatment decisions.

#### 3.1.1 Barriers among Black and African American patients

Among Black and African American patients, costs, transportation difficulties, language, and higher rates of chronic disease were identified as barriers. Limited health literacy, particularly surrounding postmenopausal bleeding, was noted by advisors. Several barriers identified among Black and African American patients involved the healthcare system and providers, including a lack of trust in HCPs and the healthcare system, implicit bias of HCPs, discrimination, and lack of representation among HCPs. Population-specific barriers included disease biology differences, including a lack of regular testing of molecular markers and an absence of prevalent molecular markers, which could affect diagnosis and treatment choice during the patient’s journey.

#### 3.1.2 Barriers among Hispanic and Latine patients

For the Hispanic and Latine population, understanding and navigating the healthcare system as well as transportation and the distance to treatment center were ranked as the greatest barriers. This was followed by provider bias, level of health literacy and lack of culturally appropriate resources, and language issues. Population-specific barriers included higher proportions of uninsured and undocumented patients and a tendency to rely on families and the community for information and decision-making.

#### 3.1.3 Barriers among AAPI patients

For the AAPI population, language issues, including specific dialects and lack of interpretation services, were ranked as being the greatest barriers, followed by lack of representation among HCPs. HCP biases and mistrust of HCPs were also identified as important barriers. Advisors noted that because the population has a bimodal income distribution, the additional needs of lower-income individuals may be masked. Population-specific barriers included a tendency to seek medical care only when health issues arise as well as strong beliefs and practices of traditional medicine. These barriers may affect awareness and recognition of symptoms and risk factors, diagnosis, and treatment choices.

#### 3.1.4 Barriers among AIAN patients

Language, lack of trust in HCPs, severe poverty, higher levels of uninsured patients, and higher levels of chronic disease, including mental health conditions, were identified as barriers among the AIAN population. Barriers identified exclusively among AIAN patients included diverse cultural belief systems and a lack of clinical infrastructure within their communities, limiting healthcare access.

#### 3.1.5 Barriers among LGBTQAI+ patients

Barriers among the LGBTQAI+ population included mistrust of the healthcare system, bias and discrimination, and higher levels of underinsured/uninsured patients. A lack of education for providers and patients about cancer risks specific to the LGBTQAI+ population was identified. Advisors said that data related to sexual orientation and gender identity have not been historically collected, contributing to this lack of knowledge surrounding risk factors, barriers to care, and behaviors. It was noted that, particularly among transgender and gender nonconforming patients, bias and discrimination are major barriers to receiving care. Population-specific barriers included a lack of support due to stigma, denial of culturally appropriate care by the healthcare system, and a lack of respect and safety. Limited research regarding the effects of androgen or testosterone therapy for transgender patients with gynecologic malignancies was also seen a barrier, as this lack of data may cause patients to make difficult decisions about whether to continue this therapy.

### 3.2 Proposed solutions for achieving equitable health care

Overall, a variety of solutions were proposed by the advisors to reduce disparities and achieve more equitable health care among all populations. Engaging patient navigators was viewed as the most impactful and achievable solution for multiple barriers across all populations (Table 2). Advisors noted that patient navigators could be used to establish trust among communities and to create culturally sensitive and safe environments for open discussions of patient needs. Training and education regarding use of patient navigators, including how to bill and submit claims for this service, was deemed critical to improve patient care. Advisors mentioned that existing training programs and certifications (see **Table S1** for examples) are not being utilized to the fullest and can be leveraged as a starting point. Additionally, advisors emphasized the need for more comprehensive training programs and certifications, such as integrating cultural sensitivity training into medical school curricula and residency programs, and the importance of face-to-face interaction in training for more effective engagement. Increased financial support for patients and cancer centers, improved HCP training, and improved community engagement were also noted as solutions that would benefit all populations.

**Table 2:** Potential solutions and recommendations for shared barriers to equitable health care.

**Most common barriers**	**Solutions and recommendations**
Costs of accessing and receiving care	• Engage patient navigators to educate on cost-related aspects of care • Cost subsidies, including for lost wages • Vouchers for groceries or meals • Assistance for patients with accessing insurance

Transportation	• Telemedicine • Financial support, including parking vouchers • Engage patient navigators to provide lists of support organizations • Reduced travel distance (eg, when feasible, use local instead of central laboratories)

Health literacy	• Education by PAGs and peers • Engage patient navigators to assist with referrals

HCP bias or cultural competence	• Engage patient navigators to assist with discussions • Sensitivity or cultural competency training • Antibias training • Involvement of family in treatment decisions • Recognition of the importance of community structure (eg, tribal or community identity)

Inadequate research among diverse populations	• Use patient navigators to educate and establish trust • Research to disaggregate racial and ethnic subgroups

Representation in clinical trials	• Use patient navigators to educate staff on how to discuss trial participation with underrepresented patients • Modified trial eligibility criteria (ie, remove unnecessary language requirements, ensure accrual represents the real-world setting) • Removal of trial enrollment barriers • Implicit bias training for clinical trial investigators and staff • Screen potential trial sites for inclusive practices • Set enrollment benchmarks based on burden of disease in the patient population

HCP, healthcare professional; PAG, patient advocacy group.

Advisors stressed the importance of tailored solutions for each population. For example, advisors recommended training for HCPs and all clinic and supportive staff to address the specific needs of the communities they serve.

#### 3.2.1 Proposed solutions specific to Black and African American patients

To address transportation issues, advisors suggested that parking passes, gas cards, shuttles, or rideshare vouchers could be provided to patients. They also encouraged the use of virtual consultations when feasible for the patient and said that patient navigators could help assess a patient’s transportation needs. Advisors noted that lack of trust and representation within health care could be addressed by increasing engagement with community advisory boards and community leaders, providing greater support for individuals from disadvantaged backgrounds, and encouraging them to enter and remain in health care. Education and training for HCPs and staff regarding the disparities faced by Black and African American patients and the impact on health outcomes were recommended. Ensuring biomarker and genetic testing for all indicated patients was also noted.

#### 3.2.2 Proposed solutions specific to Hispanic and Latine patients

Advisors recommended that providers utilize the benefits of Hispanic and Latine patients having tight-knit families to make an informed clinical care plan and involve families in decision-making. Patient navigators could assist in this process as well as foster trust and engagement with local communities. Providing interpreter services, preferably in person, and translations of educational materials were recommended to improve communication. Studies suggest that while in-person interpreters are favored by clinicians, patients equally value in-person and remote interpreters (Ruiz et al., 2025, Locatis et al., 2010); therefore, making both such modalities available would enable individualized selection, and increasing the preset appointment length accordingly to account for translation time would support equitable communication and care.

To better assist undocumented Hispanic and Latine patients in accessing care, advisors recommended that patient navigators could be a resource for information on what can be done by providers and hospitals. Advisors also said that implementing “safe zones” where patients can receive care without fear of deportation was an important step in addressing barriers in this population, along with advocating for policy changes.

#### 3.2.3 Proposed solutions specific to AAPI patients

To improve health literacy, advisors recommended using existing resources, such as those from the American Cancer Society, and local news to promote education for the AAPI community regarding cancer prevention and screening. Advisors noted that while cultural sensitivities have previously limited clinical trial enrollment and participation in preventive screening, younger generations—alongside patient navigators—can play a key role in promoting health literacy within their communities.

Advisors recommended that HCPs receive cultural competency training to better understand their AAPI patients and their hesitations, and that they partner with traditional medicine practitioners to learn and better communicate how each discipline may complement the other. Increased availability of in-person interpreter services and translated materials was also suggested.

#### 3.2.4 Proposed solutions specific to AIAN patients

Community engagement and cultural competency training were seen as achievable strategies for HCPs and pharmaceutical companies to develop trust with AIAN communities. Advisors also recommended training providers and staff to understand and incorporate diverse belief systems into Western medicine to more holistically care for their patients. Patient navigators could also help bridge potential gaps in understanding to improve patient care.

To better understand and address the impacts of poverty among AIAN patients, advisors mentioned using community organizations and programs to assist with screening for and collecting data related to social determinants of health (eg, food insecurity). Such data could then be used to assist patients and address their specific needs.

#### 3.2.5 Proposed solutions specific to LGBTQAI+ patients

Training and education for all providers as well as clinical and nonclinical staff were noted by advisors as important to addressing multiple barriers faced by LGBTQAI+ patients, including stigma, mistrust, and discrimination. Training should include how to use inclusive language when talking to patients and in all communications, as well as properly collecting and using information on a patient’s sexual orientation and gender identity in the patient’s records. Patient navigators can help to build trust with patients and encourage open discussions regarding treatment plans and transitions within cancer care. In addition, engaging community leaders and organizations was recommended as an effective strategy to strengthen trust within the community.

Advisors emphasized the importance of creating an inclusive healthcare environment for LGBTQAI+ patients. They recommended removing all gendered terminology, having gender-neutral bathrooms and gowns, and providing inclusive resources in the clinic and waiting room. Advisors also mentioned that providers should create a network of inclusive, culturally sensitive providers to whom they can refer their patients.

Advisors also expressed a need for more clinical research and data generation regarding patients who identify as LGBTQAI+. Collection of sexual orientation and gender identity data in research studies and the publication of such data would encourage more open discussion and drive education and awareness among providers and patients regarding the correlation between disparities and health outcomes in this patient community. Advisors also said that research is needed to understand the impact of testosterone use in patients with gynecologic cancers; such data will help with informed shared clinical decision-making among patients, caregivers, and physicians.

### 3.3 Barriers and solutions to equitable clinical trial participation

**Fig. 3:**
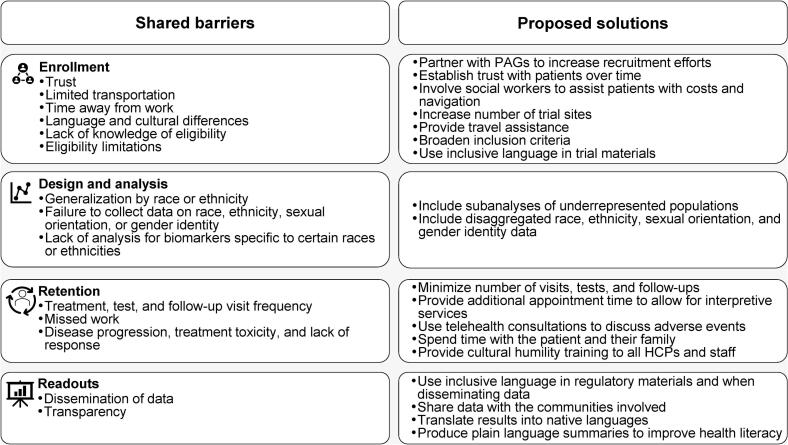
**Shared barriers and proposed solutions for equity in clinical trials.** HCP, healthcare professional; PAG, patient advocacy group.

A total of 20 barriers to equitable clinical trial participation were identified related to enrollment, design and analysis, retention, and readouts for all the population groups. Of these, 14 were common across populations (Fig. 3). Notably, some barriers to clinical trial participation overlapped with those for health care, including transportation issues, a lack of trust in providers, language barriers, and lack of cultural understanding among providers. Additionally, 6 of the barriers were found to be population-specific (Table 3).

**Table 3:** Population-specific barriers and potential solutions to clinical trial participation.

	**Barriers**	**Proposed solutions**
**Black and African American**	• Lack of regular testing and absence of prevalent molecular markers specific to the population prevent access to better targeted treatments	• Provider education and training to overcome implicit bias that results in a lack of regular biomarker testing

**Hispanic and Latine**	• Using race rather than genetic ancestry means that many patient subgroups with varying health outcomes and unstratified needs exist	• Aim to collect ancestry data as a gold standard, rather than race

**AAPI**	• Race categories provided on medical forms are insufficient for diversity of populations, resulting in many AAPI patients being placed as “other” • Lower clinical trial enrollment may occur due to uneasiness of leaving local community centers and culturally aligned health care	• Provide additional categories or open text fields for information on race • Aim to collect ancestry data as a gold standard, rather than race • Cultural humility training would support patients to leave their local community centers

**AIAN**	• Strong sense of belonging to community and culture may prevent communities from participating in clinical trials and genomic research	• Community engagement and inclusion of native/indigenous physicians and staff to integrate tribal culture into the modern clinical trial setting • Cultural humility training essential for non-native staff

**LGBTQAI+**	• Data on SOGI have not been collected during clinical trials, limiting the understanding and identification of risk factors	• Collect SOGI data for stratification and analysis

AAPI, Asian American and Pacific Islander; AIAN, American Indian and Alaska Native; LGBTQAI+, lesbian, gay, bisexual, transgender, queer (or questioning), asexual (or allied), intersex; SOGI, sexual orientation and gender identity.

Proposed solutions for equitable clinical trial participation included broadening enrollment criteria, providing additional financial support to participants, and community outreach to build trust (Fig. 3 and Table 3). Advisors noted that patient navigators could facilitate discussions with patients about clinical trial education and eligibility, assist in enrolling patients, and further community outreach efforts.

## 4 Discussion

Several key barriers to equitable health care and clinical trial participation were identified across multiple diverse populations, highlighting how alleviating these barriers has the potential to reduce healthcare disparities among many patient populations. It will also be important to address population-specific barriers to have maximal impact on patient experience and outcomes. The advisors identified barriers that overlapped between equitable health care and clinical trial participation, including transportation issues, lack of trust of HCPs, language barriers, and provider bias/cultural understanding.

Barriers were identified across all phases of the patient journey, from prevention and awareness and recognition of signs and symptoms to diagnosis, treatment, and beyond, with many barriers being relevant to multiple phases. For example, language barriers and HCP bias can influence awareness and recognition of symptoms, diagnosis, treatment decision-making, and extent of follow-up care.

Advisors highlighted the need for additional data to more fully capture the extent of health disparities in gynecologic cancer care, recommending that institutional researchers and pharmaceutical companies include and report sexual orientation and gender identity data and disaggregated data by race and ethnicity. While limited disaggregated data are currently available, these data offer a more detailed picture of health disparities, which may help develop more targeted solutions. For example, between 2001 and 2017, uterine cancer incidence increased more than 2-fold annually among Hispanic and Latine individuals compared with non-Hispanic and non-Latine White individuals in the US; differences were also seen within disaggregated Hispanic and Latine subgroups, with Dominican individuals experiencing the largest increase in diagnoses and highest proportions of advanced disease and high-risk histologic subtypes, potentially due to Afro-descendance (Reddy et al., 2024). Differences in ovarian cancer survival rates have also been observed among disaggregated Asian American populations (Lee et al., 2024).

While identifying barriers is an important step, finding and implementing impactful and pragmatic solutions to address those barriers is the only way to provide more equitable care. Engagement of patient navigators was identified by advisors as an impactful and cost-effective solution to address multiple barriers throughout the patient journey and across all populations. Patient navigators may provide decision aids, cultural messaging, and translation support to reduce the time from diagnosis to treatment initiation and connect patients with mental health services or patient support groups at the start of their patient journey (Chan et al., 2023, Pothuri et al., 2024). They can also provide information on clinical trials and assist with enrollment as well as help patients understand survivorship and improve their quality of life (Chan et al., 2023, McClung et al., 2015). Recent changes to patient navigators’ reimbursement eligibility by the Centers for Medicare & Medicaid Services now allow for the increased use of patient navigators (Centers for Medicare & Medicaid Services, 2024). The American Cancer Society’s National Navigation Roundtable provides resources to help providers and stakeholders to better understand these changes and the requirements for reimbursement (see **Table S1** for additional information) (Leadership in Oncology Navigation, 2024).

Incorporating comprehensive training programs, including antibias, antiracism, and cultural humility training, was also recommended to combat HCP bias, reduce discrimination, and create a welcoming environment for all patients. Programs such as the complimentary American Society of Clinical Oncology/Association of Community Cancer Centers (ASCO/ACCC) *Just ASK*^TM^ training program could be utilized (ASCO-ACCC, 2024). This implicit bias training program is aimed at all HCPs to help equip them in promoting diversity, inclusion, and equity in clinical trial research.

Improving diversity in clinical trials requires the engagement of multiple stakeholders, including physicians, institutions, and pharmaceutical companies. Several initiatives have been started in the U.S. to promote enrollment of diverse populations in clinical trials. These initiatives stem from both physician societies (eg, ASCO, Society of Gynecologic Oncology) (Pothuri et al., 2024, Kim et al., 2021, Oyer et al., 2022) and the U.S. government, including the U.S. Food and Drug Omnibus Reform Act 2022 (Public Law 117-328, 2022). Pharmaceutical companies have moved toward using committees to assist with study site selection and review patient enrollment data to ensure that clinical trials enroll a representative and diverse patient population. Studies have also shown that institution-level initiatives can also improve enrollment of diverse populations (Karpel et al., 2022, Boitano et al., 2023). Lastly, additional funding for gynecologic cancer research is needed (Karpel et al., 2025).

### 4.1 Limitations

While the advisory board series engaged multiple stakeholders, from researchers to oncologists to patients, it is important to recognize that additional barriers may be encountered by patients throughout their care journey that were not captured in this study. It is likely that the intersectionality of the results was not fully captured here. Patients may be members of several communities and experience barriers associated with multiple populations in ways that are different from the experiences of any single population. For example, Black and African American transgender patients may have additional or different barriers compared with White transgender patients or Black and African American cisgender patients during their care. While the perspectives of patient advisory groups and patients themselves were included in the present study, the majority of participants were clinicians and researchers; future studies incorporating additional discussion groups and surveys with a patient focus may be warranted to complement and extend the present findings.

## 5 Conclusion

Common barriers to care were identified across populations, including cost, transportation, level of health literacy, and provider biases. However, more of the barriers identified were population specific, affirming the need for continued consultation and discussions with members of specific communities and with individuals to address specific barriers and enact effective solutions. Involvement of patient navigators was identified as a highly impactful and pragmatic solution for breaking multiple barriers across various diverse populations.

## Data sharing statement

GSK makes available anonymized individual participant data and associated documents from interventional clinical studies that evaluated medicines, upon approval of proposals submitted to: https://www.gsk-studyregister.com/en/. For other types of GSK-sponsored research, study documents without patient-level data, and clinical studies not listed, please submit an inquiry through the website.

## Role of funding source

This study was funded by GSK. Medical writing support was funded by GSK. GSK was involved in the design, data analysis and interpretation, and writing of the report. All authors, including those employed with GSK, were involved in the decision to submit the manuscript for publication.

## CRediT authorship contribution statement

**Bhavana Pothuri:** Writing – review & editing, Writing – original draft, Conceptualization. **Michele Muir:** Writing – review & editing, Visualization, Project administration, Methodology, Data curation, Conceptualization. **Jean Hurteau:** Writing – review & editing, Supervision, Methodology, Formal analysis, Conceptualization. **John Farley:** Writing – review & editing. **Michelle D.S. Lightfoot:** Writing – review & editing. **Summer Dewdney:** Writing – review & editing. **Tara Castellano:** Writing – review & editing. **John K. Chan:** Writing – review & editing. **Sharad Ghamande:** Writing – review & editing. **Al Asante-Facey:** Writing – review & editing. **Marina Stasenko:** Writing – review & editing. **B.J. Rimel:** Writing – review & editing. **Electra D. Paskett:** Writing – review & editing.

## Declaration of competing interest

The authors declare the following financial interests/personal relationships that may be considered as potential competing interests: **BP** reports grants or contracts from Tesaro/GSK, Merck, AstraZeneca, Karyopharm Therapeutics, Sutro Biopharma, Incyte, Toray, VBL Therapeutics, InxMed, Agenus, Seagen Inc, NRG Oncology, Duality Bio, Clovis Oncology, Roche/Genentech, Mersana, Celsion/Imunon, I-Mab, Takeda, Onconova, Celgene, Novocure, ImmunoGen, Eisai, Acrivon, Xencor, and Alkermes; consulting fees from Tesaro/GSK, AstraZeneca, Merck, ImmunoGen, GOG Foundation, Seagen Inc, Lilly, Eisai, Signatera, Celsion, Sutro Biopharma, Imvax Inc, Incyte Corporation, InxMed, Onconova Therapeutics, R Pharm, Regeneron, and Duality Bio; travel support from GOG Partners; and data safety monitoring board or advisory board participation for Sutro, AstraZeneca, GOG Foundation, Celsion/Imunon, Toray, InxMed, Onconova, Imvax, I-Mab, Tesaro/GSK, Merck, Mersana, Nuvation, and BioNTech. **MM** and **JH** are employees of GSK plc. **JF**, **SD**, **TC,** and **MS** have nothing to disclose. **MDSL** reports other financial or nonfinancial interests with GSK (disparities, advisory boards, and OncLive presentations). **JKC** reports consulting fees from AbbVie, Agenus, AstraZeneca, Daiichi Sankyo, Eisai, GSK, Genmab, Seagen Inc, ImmunoGen, Karyopharm, Merck, Mersana, MTTI, Myriad, Pfizer, and Roche and travel support from NRG/GOG. **SG** reports research funding from AbbVie, Advaxis, Akeso Biopharma, Aravive, Astellas Pharma, BMS, Clovis Oncology, Ellipses Pharma, Genentech, GSK, Incyte, Jounce Therapeutics, Merck, Merck Serono, Roche, Seagen Inc, Takeda, and Tesaro; consulting fees from Seagen Inc; and honoraria from Eisai, Tesaro, and GSK. **AA-F** reports consulting fees from Merck; travel support from GSK; and data safety monitoring board or advisory board participation with GSK and AstraZeneca. **BJR** reports consulting fees with GSK and advisory board participation with GSK, Merck, ImmunoGen, and AstraZeneca. **EDP** reports consulting fees from GSK; research funding from Genentech, Guardant Health, Merck, and Pfizer; grant funding from AstraZeneca; and advisory board participation with Merck.
