# Supplementary material for: Identifying and breaking barriers: Addressing disparities in the care of patients with gynecologic cancers
Source: Gynecol Oncol Rep. 2025 Jun 30;60:101799. doi: 10.1016/j.gore.2025.101799 (PMC12272470; doi:10.1016/j.gore.2025.101799)
Supplement: Supplementary Data 1 [file mmc1.docx]

# Supplemental material

# Supplemental methods

## GSK Endometrial Cancer Healthcare Disparities Asynchronous Advisory Board - Touchpoint #1 Survey Questions

## GSK Endometrial Cancer Healthcare Disparities Asynchronous Advisory Board - Touchpoint #2 Survey Questions

## GSK Endometrial Cancer Healthcare Disparities Asynchronous Advisory Board - Touchpoint #3 Survey Questions

# Supplemental tables and figures

## Table S1. Additional information on currently available trainings and certifications

## Figure S1. Longitudinal engagement plan timeline

## Figure S2. Sample agenda from live advisory board

## Figure S3. Breakdown of participants by touchpoint

# Supplemental methods

## GSK Endometrial Cancer Healthcare Disparities Asynchronous Advisory Board - Touchpoint #1 Survey Questions

**Section 1 - Your Clinical Practice**

In this section, we have a few questions about your practice.

Q1. Your Clinical Practice

Based on the typical patients you encounter in your clinical practice, please rank the following patient groups in order of prevalence, with “1” being the most common.

Please note: when ranking, each number may only be used once.

1. Race/ethnicity
   1. White
   2. Black
   3. Hispanic
   4. Asian or Pacific Islander
   5. Indigenous
   6. Other
2. Language
   1. Native English speakers
   2. English as a second language (ESL)
   3. Non-English speakers
3. Residence
   1. Resides in a rural area
   2. Resides in an urban center or its suburbs
4. LGBTQIA+ community
   1. Lesbian/bisexual
   2. Transgender
   3. Non-binary

Q2. Socioeconomic Factors

In your experience, are there any socioeconomic factors that may negatively influence the time and approach to diagnosis, treatment, and follow-up for patients with endometrial cancer (assuming similar clinical/tumor characteristics)? If yes, please elaborate.

Q3. Cultural Considerations

1. In your experience, are there any cultural factors that may negatively influence the time and approach to diagnosis, treatment, and follow-up for patients with endometrial cancer (assuming similar clinical/tumor characteristics)? If yes, please elaborate.
2. Do language barriers impact patients’ ability to get timely diagnosis and treatment for endometrial cancer? Please explain your answer.
3. Do you have experience with interpreters (in-person or virtually) as part of your patient consultations? If so, please share how often you communicate with interpreters in a typical month and describe your experience.

**Section 2 - Racial Disparities**

In this section, we would like you to review and answer questions on recent congress abstracts that presented healthcare disparities in endometrial cancer.

Q1. Abstract Review: Anastasio et al. (2023)

Please review *Healthcare access dimensions and uterine cancer survival: A National Cancer Database study* by Anastasio et al. (2023).

1. Please share your thoughts on the study results, conclusions, and implications.
2. What are the key takeaways from this study?
3. Do the study’s findings match what you see in your clinical practice?

Q2: Abstract Review: Anastasio et al. (2023) cont’d

Anastasio et al. concluded that “healthcare access affordability predicts survival but does not fully explain racial disparities in survival rates of patients with advanced stage uterine cancer. Non-Hispanic Black patients are more likely to have aggressive disease, receive chemotherapy alone, and have worse survival than Non-Hispanic White patients regardless of pathogenic subtype. Investigating additional healthcare access dimensions may be critical to addressing disparities in uterine cancer.”

1. Aside from healthcare access affordability, what other factors may contribute to the worse survival rates among Black patients with uterine cancer? Please explain why these factors may contribute to worse survival.
2. How do you believe these gaps could be addressed? Please be specific.
3. What are some specific research questions that would help address these disparities and/or help identify their causes?

Q3. Abstract Review: Xu et al. (2023)

Please review *Racial and ethnic disparities in quality of diagnostic evaluation for uterine cancer among Medicare patients* by Xu et al. (2023).

1. Please share your thoughts on the study results, conclusions, and implications.
2. What are the key takeaways from this study?
3. Do the study’s findings match what you see in your clinical practice?

Q4. Abstract Review: Xu et al. (2023)

Xu et al. found that:

- A larger proportion of Black vs. white patients did not receive guideline-recommended diagnostic procedures
- The time from abnormal uterine bleeding presentation to first diagnostic procedure was longer for Black vs. white patients
- Black patients were more likely than white patients to be diagnosed in the late stage rather than early (localized) stage.

The authors concluded that there were racial/ethnic disparities in the quality of diagnostic evaluation received by Medicare patients with uterine cancer, which may partially explain Black-white differences in their stage at diagnosis.

How can these racial disparities be overcome? For example, is there a need for more research; provider, patient, or public education; awareness campaigns; health literacy initiatives; digital tools; or community outreach initiatives related to diagnosis? In your answer, please be as specific as possible.

Q5. Abstract Review: Chapman-Davis et al. (2022)

Please review *Identifying disparities in gynecologic cancer: Results and analysis from a patient preference survey* by Chapman-Davis et al. (2022) and provide your thoughts by annotating the paper.

To annotate the document:

1. Click the button below to launch the viewer
2. In the top toolbar, click "Point Comment" to enter a comment at a specific point or "Area Comment" to select a block of text to comment on.
3. Add your comments in the sticky note that appears after you click.

All annotations will be visible. We encourage you to review and reply to the comments of the other advisors. Your changes will be saved as you go.

Q6. Abstract Review: Chapman-Davis et al. (2022) cont’d

Chapman-Davis et al. found that underrepresented (non-white) patients felt less prepared to discuss treatment-related issues compared to white patients. Moreover, a large proportion of all patients with ovarian cancer were not informed and/or aware about genetic testing, and approximately a third of participants were not confident in their ability to interact with their provider to improve their outcomes. According to the authors, these results highlight opportunities to enhance health care provider education and community outreach to reduce gaps in care delivery.

1. In your opinion, is there a need for more or better healthcare provider education to address these issues? If *yes*, what are the current gaps that education can help overcome? If *no*, please explain why not.
2. How do you see community outreach playing a role in reducing care gaps in care delivery?
3. How can GSK best partner with healthcare providers and patients to support educational and/or community outreach initiatives?

Q7. Abstract Review: Giaquinto et al. (2022)

Please review *The Changing Landscape of Gynecologic Cancer Mortality in the United States* by Giaquinto et al. (2022).

1. Do these results reflect what you are seeing in your clinical practice?
2. What are your key takeaways from the research letter?
3. Is there anything about these results that surprised you?

**Section 3 - Underserved Communities**

In this section, we have a few final questions on underserved communities (e.g. LGBTQIA+ and Indigenous populations).

Q1. LGBTQIA+ Health Disparities

Members of the LGBTQIA+ community carry a disproportionate cancer burden, with higher cancer incidence, later-stage diagnosis, and greater mortality rates across many cancer types. The chart below illustrates major health discrepancies between heterosexual, lesbian/gay/bisexual (LGB), and transgender individuals. In addition, women/people with a uterus who have never been pregnant or have never taken oral contraceptives are at higher risk of endometrial cancer.

**
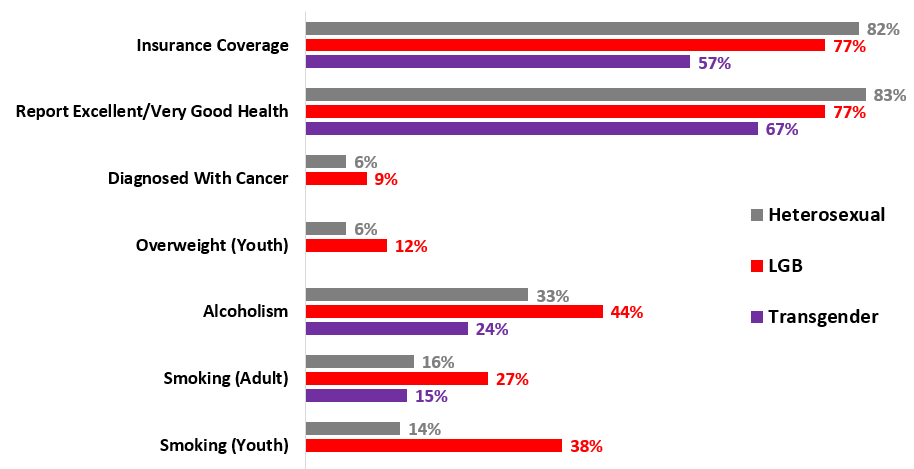
**

1. Why do you think patients belonging to the LGBTQIA+ community face a disproportionate cancer burden (i.e., higher cancer incidence, later-stage diagnosis, and greater mortality rates)?
2. What can be done to alleviate the disproportionate cancer burden in the LGBTQIA+ population?
3. With the percentage of patients identifying as belonging to the LGBTQIA+ community increasing and the eventual aging population, how can we support those assigned female at birth with gynecologic malignancies? For example, what could be done to improve the time to diagnosis and survival rates in this population?

Q2. Indigenous Populations

Indigenous people often have higher incidence and mortality rates of cancers related to exposure to tobacco, alcohol, poor diet, physical inactivity, high BMI, and diabetes mellitus than non-Indigenous people living in the same countries, although cancer patterns vary from country to country. Further, survival after diagnosis is lower, and its improvement is slower in Indigenous populations, suggesting that Indigenous populations have not benefited equally from advances in early detection and treatment. The chart below illustrates the relative risk of cancer-specific mortality for Indigenous vs. non-Indigenous females by country and tumor site.


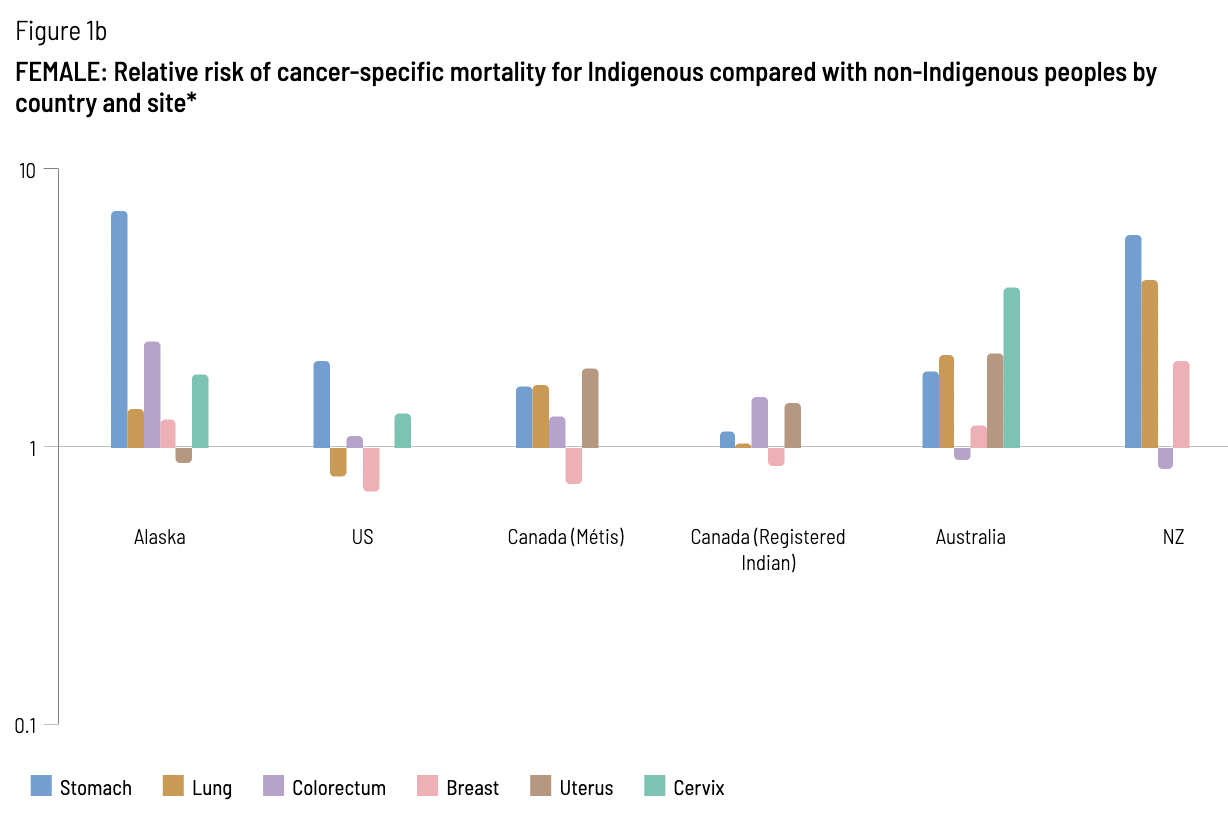


1. Why do you think Indigenous people face a disproportionate cancer burden (i.e., higher cancer incidence, later-stage diagnosis, and greater mortality rates) in the US?
2. What can be done to alleviate the disproportionate cancer burden in Indigenous populations across the US?
3. For endometrial cancer patients specifically, what can be done to improve the time to diagnosis and survival rates among Indigenous populations in the US?
   d) Among Indigenous populations with a strong sense of community, how can we best explain and raise awareness for genetic testing of the tumor and/or familial testing if there is a concern for Lynch Syndrome?

Q3. Equal Access to Care for Underserved Communities

In this question, we would like your feedback on drivers and barriers for underserved communities when trying to access care. To the image below, please add a car for each strategy that you believe would DRIVE equal access to routine healthcare, cancer screening, and oncologist/multidisciplinary care for patients from underserved communities. Please also add a stop sign to represent each BARRIER that patients from underserved communities face when trying to access care.

1. To add a car (driver) or stop sign (barrier), click the appropriate button at the top of the image.
2. In the box which appears:
   - Enter a brief description of your driver/barrier in the “Title” field.
   - Provide a detailed explanation of your driver/barrier in the “Description field”.
   - Click “Submit”.
3. Drag your driver/barrier to the image.
4. Repeat steps 2 and 3 as applicable to add more drivers and barriers to the map.
5. To edit your entry, click the pencil icon on the right side of your driver/barrier and make any changes needed to the title or description before clicking “Submit”.
6. To view the details of the drivers and barriers placed on the map by other advisors, simply hover over the cars and stop signs and the details will appear.

Q4. Role of Telemedicine

Please review *Variation in telemedicine usage in gynecologic cancer: Are we widening or narrowing disparities?* by Andriani et al. (2022).

The authors reported regional disparities across cancer types and oncology settings in terms of telemedicine use. They concluded that expanding access to telemedicine may improve racial and geographic disparities in gynecologic cancer.

1. Do you agree with this conclusion? Why or why not?
2. Where do you see telemedicine providing the most value to your patients with endometrial cancer?
3. What are some potential reasons for the limited use of telemedicine among patients with gynecologic cancers (in general and in patients from underserved communities)?
4. How could these barriers be overcome?

Q5. Clinical Trial Access

Please review the summary of *Leveling the playing field: Identifying barriers and patterns of endometrial cancer clinical trial enrollment for underrepresented groups* by Lee et al. (2022) below.

The authors set out to identify barriers and patterns of clinical trial enrollment for patients with endometrial cancer from underrepresented groups. They found that one in ten studies in gynecologic oncology did not allow non-English speakers to enroll in clinical trials. Allowing access to clinical trials regardless of primary language may decrease barriers to enrollment for patients historically underrepresented in trials. In addition, improving demographic data reporting is imperative and can identify additional barriers to trial enrollment.

1. Thinking of patients with endometrial cancer in your community, what barriers are they facing in terms of enrolling in clinical trials?
2. Do the barriers differ for different patient populations? In your response, please be as specific as possible.

**Section 4. Conclusion**

We very much appreciate your time and commitment to this initiative. We are striving to offer a valuable experience to you, our trusted advisor, and we would appreciate your feedback on the virtual/online format of this advisory board. Thank you in advance for your feedback – it is appreciated and will be used to ensure the success of future touchpoints.

Q1. User Experience

Please rate your overall user experience with the portal (1=not very user-friendly, 5= extremely user-friendly)

Q2. Overall Experience

In the context of this virtual/online advisory board touchpoint, please share your overall thoughts regarding your experience using this virtual/online format (e.g. was the experience efficient and user friendly, have you used similar platforms previously, was the amount of time allocated sufficient, was the level of interactivity appropriate, etc.).

Q3. Feedback

Can we publish your name and your feedback about the online advisory board experience (from the question above), e.g. on the Impetus Digital Website?

1. Yes, you can publish my name with my feedback.
2. No, I prefer my feedback to remain anonymous.

Q4. Additional Discussion

Given what was discussed during this activity, what, if any, outstanding topics or content do you think would be worthy of discussion in an advisory board capacity in the future?

Q5. Continued Participation

Would you like to continue participating in similar virtual/hybrid activities and discuss the topic(s) indicated above?

If No, what other kinds of virtual initiatives, if any, would you be interested in participating in?

If Yes, how soon would you like to meet again?

1. Within 1 month
2. Within 3 months
3. Within 6 months
4. Within 9 months
5. Within 12 months
6. Other

If Yes, how often would you like to participate in these virtual activities?

1. Every month
2. Every 2 months
3. Quarterly
4. 2-3 times a year
5. Other

Q6. Updates

Would you like to receive updates on how we have leveraged your insights?

Q7. Preferences

What is your preference for future advisory board meetings?

1. In-person Meetings
2. Hybrid Meetings (In-person Meetings with the choice to attend virtually)
3. Web Meetings
4. Virtual Asynchronous Activities
5. Combination of Hybrid/Web Meetings and Virtual Asynchronous Activities
6. Other

Q8. Benefits of Virtual Meetings

In your opinion, what is the biggest benefit(s) of virtual vs. in-person meetings? [select all that apply]

- Convenience/flexibility
- No need to take time off work/away from patients
- Low/no environmental impact
- More diverse voices can be heard
- No language barriers
- N/A - I prefer in-person meetings over virtual meetings
- Other

Q9. Final Thoughts

Are there any final thoughts you would like to share with us?

## GSK Endometrial Cancer Healthcare Disparities Asynchronous Advisory Board - Touchpoint #2 Survey Questions

**Section 1 – Communication with Patients**

In this section, we would like to better understand some general considerations around communication with patients about clinical trials.

Q1. HCP Awareness

1. How do you become aware of clinical trials that your patients could be enrolled in?
2. What recommendations do you have for keeping physicians up to date on the latest clinical trials that could benefit your patients? Please be specific.

Q2. Patient Awareness

1. In your experience, how do patients typically become aware of clinical trials? e.g. from patient advocacy groups, HCPs, online searches, etc.
2. Do you have any recommendations for increasing patient awareness of clinical trials? Please be specific.

Q3. Presentation of Opportunity to the Patient

1. Who typically presents an endometrial cancer clinical trial to the patient and explains the eligibility criteria (e.g. medical oncologist, gynecological oncologist, patient navigators, etc.)?
2. At what point in the patient journey does this discussion usually occur?

Q4. Patient Education

1. Are there specific education needs for patients with endometrial cancer?
2. What patient resources do you find are most helpful to patients? Please be specific.

Q5. Patient Enrollment

1. In your experience, what are the most important services that support patient enrollment in clinical trials?
2. Are there any services not currently available that you think would effectively support patient enrollment in trials?

**Section 2. Patient Motivation**

In this section, we have a few questions about patient motivations and reservations for participating in clinical trials.

Q1. Patient Motives

1. What do you perceive are the motives for patients to agree to participate in clinical trials? Please be specific.
2. What do you perceive are the motives for patients to decline participation in clinical trials, i.e. the most common challenges that prevent patients from considering clinical trials? Please be specific.
3. Are there ways to design a trial that is more likely to interest patients and offer an optimal experience to patients? Please be specific.

Q2. Patient Populations

1. Have you observed more reservations from some patients/patient populations in accepting / declining clinical trials? Please explain in detail.
2. What could be done to make trial participation more feasible for these patients? Please be specific.
3. Please describe in detail your experience enrolling diverse patient populations in clinical trials.

**Section 3. Geographical Considerations**

In this section, we would like to learn about the geographical considerations that impact patient participation in clinical trials.

Q1. Geography of Disease and Trials

1. In your experience, does endometrial cancer vary geographically?
2. Is the geography of clinical trials aligned with the geography of disease?
3. From a geographical standpoint, do you perceive that endometrial cancer trials are available to most patients, or are there regions that are being missed?

Q2. Transportation

1. How does time and travel influence a patient’s decision to participate in a clinical trial?
2. Do most patients have access to endometrial cancer trials in their local area?

Q3. Telehealth

Please comment on how the inclusion of telehealth or other virtual tools could expand the pool of potential patients, such as those that live in rural or remote areas.

**Section 4. Clinical Considerations**

In this section, we would like to better understand how clinical considerations affect patient involvement in clinical trials.

Q1. Inclusion Criteria

1. Which clinical trial inclusion criteria do you feel are most appropriate as inclusion criteria for an endometrial cancer trial (for example: age, measurable disease, histological confirmation, cancer stage, performance status)? Please be specific and explain your rationale.
2. Which clinical inclusion criteria do you feel create a barrier to patients’ participation in endometrial cancer clinical trials?

Q2. Exclusion Criteria

1. In your experience, what are the most common exclusion criteria that prevent patients who might otherwise be suitable candidates for a trial from qualifying for endometrial cancer clinical trials (for example: age, performance status, previous treatments, prior malignancies, co-existing medical conditions? Please be specific and explain your rationale.
2. Based on your responses above, what do you think would be the most effective ways to address these issues. Please explain your rationale.

**Section 5: Optimizing Participation in Trials**

In this section, we have a few final questions on how patient participation in clinical trials could be optimized.

Q1. Patient Needs

1. Where have past clinical trials fallen short of meeting the needs of your patients with endometrial cancer?
2. Does this have to do with the design of the trial, the schedule of assessments, etc.? Please be specific.

Q2. On-Trial Support Offerings

1. In your experience, what on-trial support offerings have the biggest impact on a positive patient experience in a clinical trial?
2. Are there any supports not currently offered in clinical trials that you feel should be offered in order to optimize the patient experience?

Q3. Patient Retention

1. For what reasons do patients typically stop participating in a trial?
2. Do you have any advice for retaining patients on trials (for example, specific means of support to assist with patients staying in the trial)?

**Section 6. Conclusion**

We very much appreciate your time and commitment to this initiative. We are striving to offer a valuable experience to you, our trusted advisor, and we would appreciate your feedback on the virtual/online format of this advisory board. Thank you in advance for your feedback – it is appreciated and will be used to ensure the success of future touchpoints.

Q1. User Experience

Please rate your overall user experience with the portal (1=not very user-friendly, 5= extremely user-friendly)

Q2. Overall Experience

In the context of this virtual/online advisory board touchpoint, please share your overall thoughts regarding your experience using this virtual/online format (e.g. was the experience efficient and user friendly, have you used similar platforms previously, was the amount of time allocated sufficient, was the level of interactivity appropriate, etc.).

Q3. Feedback

Can we publish your name and your feedback about the online advisory board experience (from the question above), e.g. on the Impetus Digital Website?

- 1. Yes, you can publish my name with my feedback.
  2. No, I prefer my feedback to remain anonymous.

Q4. Additional Discussion

Given what was discussed during this activity, what, if any, outstanding topics or content do you think would be worthy of discussion in an advisory board capacity in the future?

Q5. Updates

Would you like to receive updates on how we have leveraged your insights?

Q6. Final Thoughts

Are there any final thoughts you would like to share with us?

## GSK Endometrial Cancer Healthcare Disparities Asynchronous Advisory Board - Touchpoint #3 Survey Questions

**Current State**

Before proceeding to the questions in this touchpoint, we are interested in your thoughts on the current state of access to care and treatment outcomes for patients with endometrial cancer. In the questions that follow, we will explore this topic further.

In the bullseye figure below, please click on one of the 4 rings.

**Scale (from outer ring to inner ring):**

- Misses the mark
- Needs improvement
- Almost perfect
- Nails it (access to care and treatment outcomes are optimized/equitable)

Please explain your response.

**Section 1: Healthcare System and Advocacy**

Q1. Healthcare System

1. What systemic factors within the healthcare system positively impact equitable access to care and treatment outcomes for patients with endometrial cancer? Please explain your response.
2. What systemic factors within the healthcare system have a negative impact on equitable access to care and treatment outcomes for patients with endometrial cancer? Please explain your rationale.
3. Are there policy, regulatory, or other changes that you believe could help bridge the gaps you mentioned in part b)? Please be specific.

Q2. Advocacy

1. Are you aware of any patient advocacy efforts that have proven effective in ensuring equitable access to care and treatment outcomes for patients with endometrial cancer? Please specify.
2. How can GSK collaborate with advocacy groups to amplify patient advocacy efforts and improve affordability, access, and quality of care for endometrial cancer patients?

Q3. Collaborative Opportunities

1. In what ways can pharmaceutical companies collaborate with HCPs to address care access issues?
2. Are there partnership models or strategies that could enhance patient access to care and support?

**Section 2: Barriers and Solutions**

Q1. Current Challenges - Patients

1. What are the primary barriers patients with endometrial cancer face in accessing care or treatments?
2. How can awareness and sensitivity to cultural differences impact access to care and treatment outcomes among diverse populations affected by endometrial cancer?
3. How can this situation be improved? What can GSK do to be part of the solution or help find solutions? Please be specific.

Q2. Current Challenges – Healthcare Providers

1. What challenges do healthcare providers face in delivering equitable care to patients with endometrial cancer, and ensuring consistent treatment outcomes for all patients?
2. How can this situation be improved? What can GSK do to be part of the solution or help find solutions? Please be specific.

**Section 3: Education**

Q1. Patient Education and Support

What educational resources or support programs would be most beneficial for patients with endometrial cancer as they navigate their journey to improve understanding, compliance, and overall treatment outcomes? Please specify the recommended topics (e.g. diagnosis, treatment options, side effects, post-treatment care, etc.?) as well as how this education would be best delivered to patients.

Q2. Recognizing Symptoms of Endometrial Cancer

1. Please share your thoughts on recognizing symptoms of endometrial cancer as an educational topic for at-risk populations. In your opinion, is this an important topic? Why or why not?
2. Are cultural differences/sensitivities a barrier to symptom recognition or acknowledgement of abnormal symptoms? How could this barrier be overcome?

Q3. Patient Engagement

1. What approaches or initiatives are you aware of (outside of the endometrial cancer landscape) that have proven effective in engaging patients from underserved or marginalized communities?
2. How can these be expanded or replicated in the endometrial cancer landscape to enable equitable access to care and treatment outcomes?

Q4. HCP Education

1. What specific educational resources, programs, or platforms do you find most effective in staying up to date on advancements in endometrial cancer care and treatment?
2. What are the specific areas/topics within the endometrial cancer space where additional training or educational support is needed to further enable healthcare providers to improve patient outcomes? Please explain your rationale.

Q5. HCP Education (cont’d)

Thinking of a learning program you have participated in that was particularly successful, please outline what made it so successful for you.

Q6. Data Sharing

What methods do you find most effective for pharmaceutical companies to share new data with you (e.g. research findings), and why? Please be specific and explain your rationale.

**Section 4: Future Steps**

Q1. Joint Research and Development

What potential research projects or areas of development could benefit from joint efforts between GSK and healthcare providers to enable equitable access to care and treatment outcomes for endometrial cancer patients?

Q2. Technology and Innovation

1. Please share your thoughts on how technology and innovation could be leveraged to improve patient access to care and treatment outcomes. Are there specific tools or solutions that you believe could streamline care and/or improve patient outcomes?
2. How can GSK collaborate to support these innovations?

**Section 5. Conclusion**

We very much appreciate your time and commitment to this initiative. We are striving to offer a valuable experience to you, our trusted advisor, and we would appreciate your feedback on the virtual/online format of this advisory board. Thank you in advance for your feedback – it is appreciated and will be used to ensure the success of future touchpoints.

Q1. User Experience

Please rate your overall user experience with the portal (1=not very user-friendly, 5= extremely user-friendly).

Q2. Overall Experience

In the context of this virtual/online advisory board touchpoint, please share your overall thoughts regarding your experience using this virtual/online format (e.g. was the experience efficient and user friendly, have you used similar platforms previously, was the amount of time allocated sufficient, was the level of interactivity appropriate, etc.).

Q3. Feedback

Can we publish your name and your feedback about the online advisory board experience (from the question above), e.g. on the Impetus Digital Website?

- 1. Yes, you can publish my name with my feedback.
  2. No, I prefer my feedback to remain anonymous.

Q4. Additional Discussion

Given what was discussed during this activity, what, if any, outstanding topics or content do you think would be worthy of discussion in an advisory board capacity in the future?

Q5. Updates

Would you like to receive updates on how we have leveraged your insights?

Q6. Final Thoughts

Are there any final thoughts you would like to share with us?

# Supplemental tables and figures

# Table S1. Additional information on currently available trainings and certifications

| **Organization** | **Details** | **Additional information and resources** |
| --- | --- | --- |
| American Cancer Society National Navigation Roundtable | - Coalition of more than 200 organizations that aims to advance patient navigation to eliminate barriers to quality care, reduce disparities, and promote health equity throughout patients’ cancer journeys - Funds patient navigators and patient navigation programs, develops training curricula, and provides training and credentialing for professional navigators | Additional information on training offered by the National Navigation Roundtable: <https://www.cancer.org/health-care-professionals/resources-for-professionals/patient-navigator-training.html>  Additional resources from the National Navigation Roundtable:  <https://navigationroundtable.org/resource-center/> |
| Centers for Medicare & Medicaid Services (CMS) 2024 Physician Fee Schedule | - Allows reimbursement for nonclinical navigation services for patients with a “principal illness,” including cancer | Additional information on CMS reimbursement for patient navigation services:  <https://www.cancer.org/content/dam/cancer-org/online-documents/en/pdf/brochures/acs-lion-pin-payment-faq-07-22-24.pdf>  <https://www.cms.gov/files/document/health-related-social-needs-faq.pdf> |
| Patient Navigation & Community Health Worker Training (PNCT) | - Provides a variety of courses and curricula for both new and experienced patient navigators | <https://patientnavigatortraining.org/> |
| National Committee for Quality Assurance (NCQA) | - NCQA’s Patient Centered Medical Home model of care recognizes the value of navigators in care quality | <https://www.ncqa.org/programs/health-care-providers-practices/patient-centered-medical-home-pcmh/> |
| COLORS Training | - An interactive web-based LGBTQAI+ cultural sensitivity training designed for oncologists, aiming to educate healthcare providers on how to better serve LGBTQAI+ patients | https://www.sciencedirect.com/science/article/abs/pii/S0738399118305317?via%3Dihub |
| Welcoming Spaces Webinar Series | - Created in partnership with SGO to target gynecologists and gynecologic oncologists who treat LGBTQAI+ people, covering topics from making office and staff welcoming to treating patients with ovarian cancer | https://connected.sgo.org/content/welcoming-spaces-series |
| SGO Cultural Humility Training | - Eight-session series available on the SGO website and focused on cultural humility and sensitivity training for healthcare providers; includes certification upon completion | https://www.sgo.org/welcomingspaces/ |
| ASCO-ACCC Just Ask! Program | - A program designed to address implicit bias in clinical trial enrollment, emphasizing the importance of asking all eligible patients to participate in clinical trials | <https://courses.accc-cancer.org/products/just-ask-increasing-diversity-in-cancer-clinical-research> |

# Figure S1. Longitudinal engagement plan timeline. AAPI, Asian American and Pacific Islander; HCP, healthcare professional; LGBTQAI+, lesbian, gay, bisexual, transgender, queer (or questioning), asexual (or allied), intersex; T, advisory board touchpoint.

**
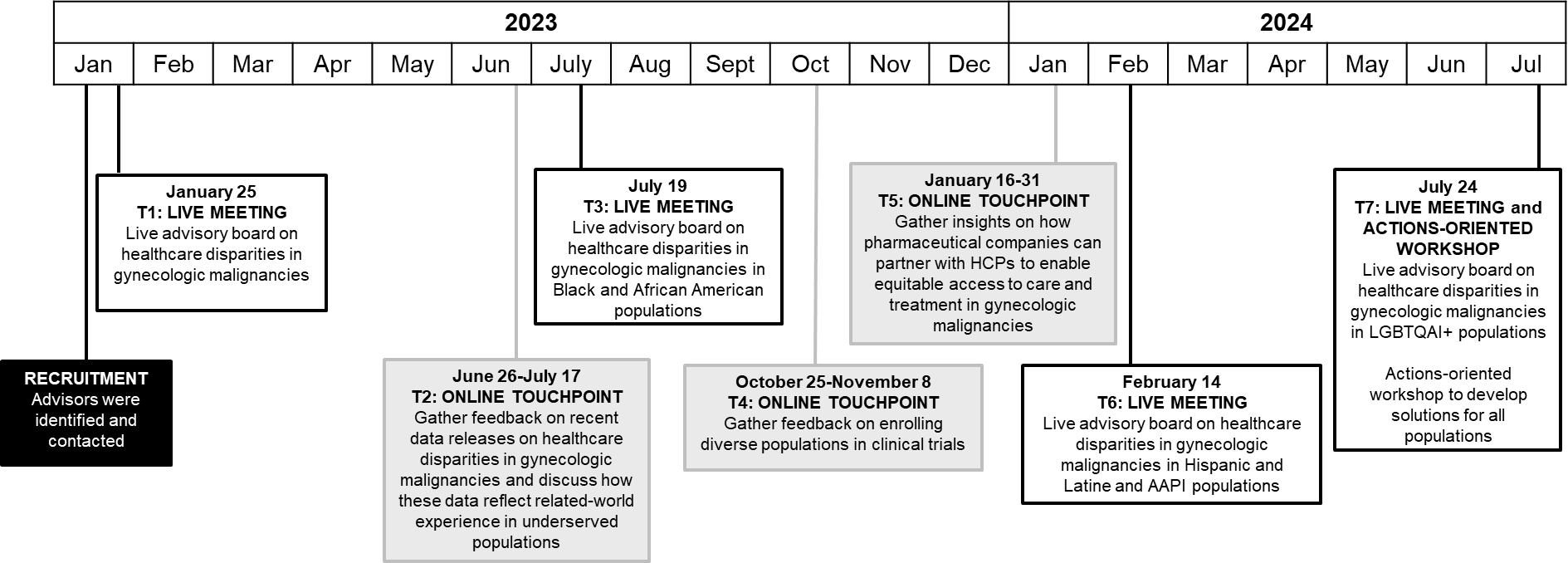
**

# Figure S2. Sample agenda from live advisory board.


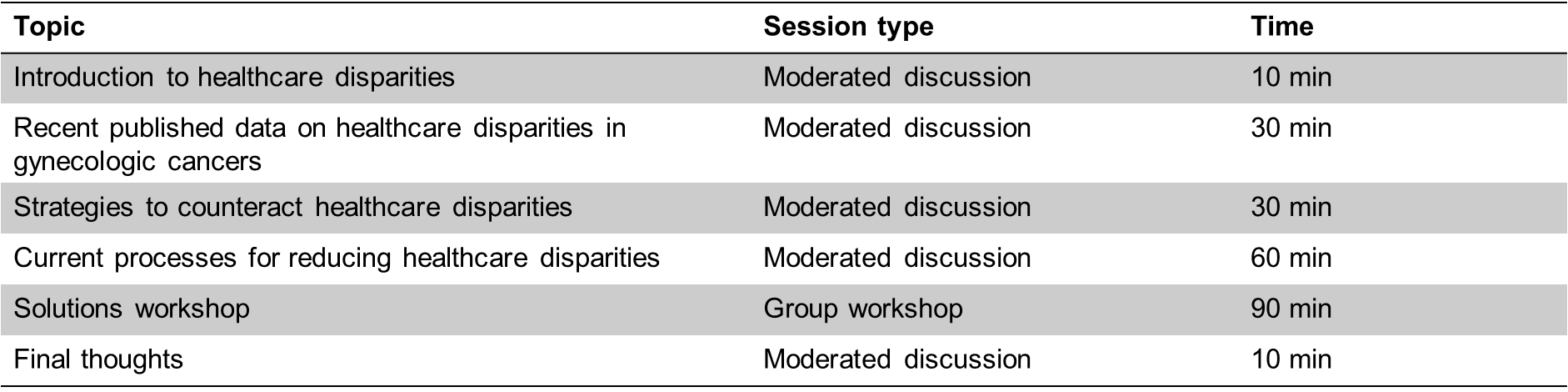


# Figure S3. Breakdown of participants by touchpoint.^a^ PAG, patient advocacy group; T, advisory board touchpoint.


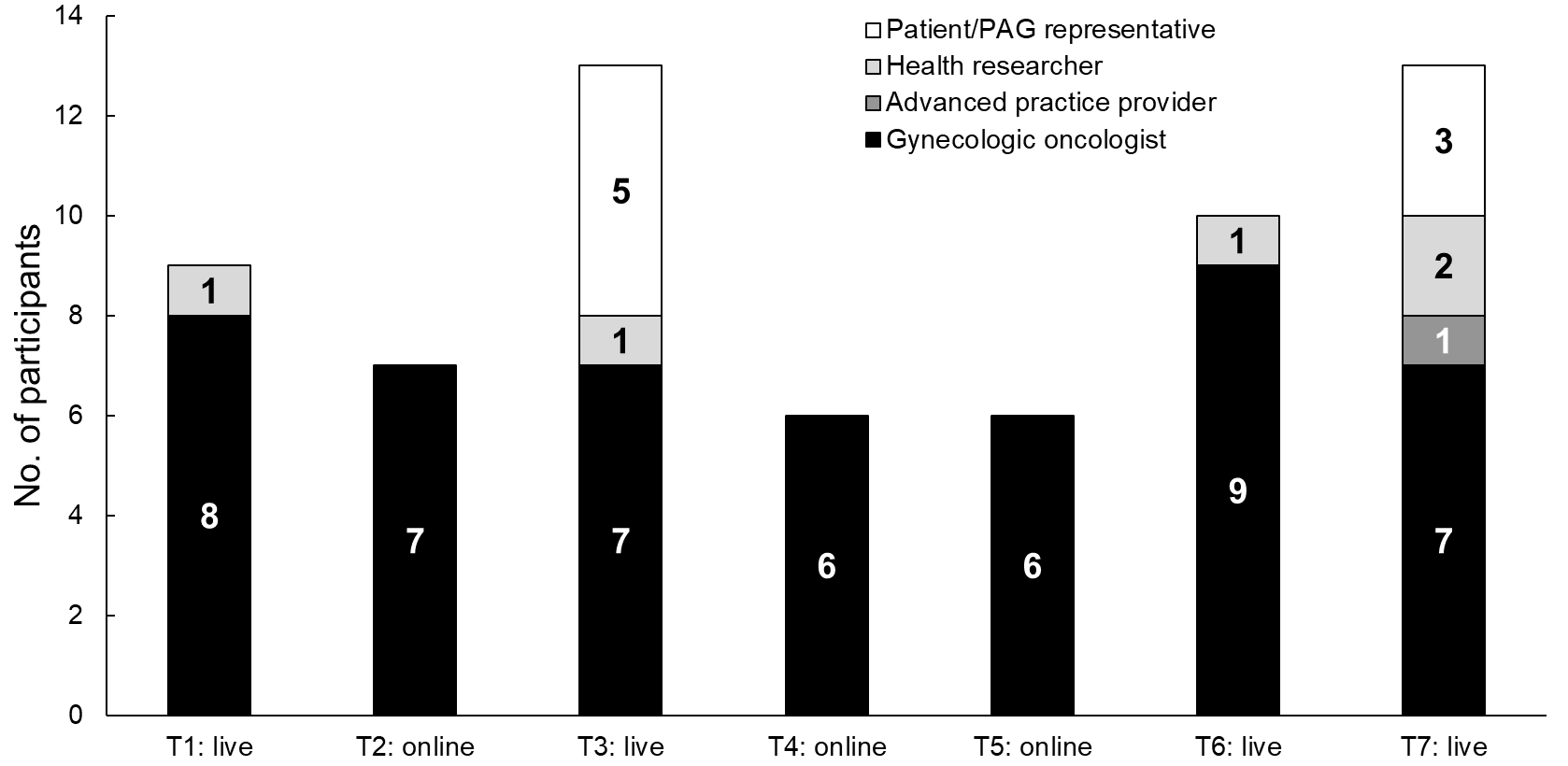


^a^T3, T6, and T7 consisted of 2 sessions each. The number of participants differed between sessions with ≤12 attendees in each session of T6 and T7. T3 received an exception to include 13 participants in the second session.
